# Revealing transcriptomic responses in *Escherichia coli* during early antibiotic exposure

**DOI:** 10.1128/msystems.01584-25

**Published:** 2026-03-10

**Authors:** Yuan Yuan, Ying Hefner, Richard Szubin, Jaemin Sung, Bernhard O. Palsson

**Affiliations:** 1 Shu Chien-Gene Lay Department of Bioengineering, University of California San Diego 8784, La Jolla, California, USA; 2 Bioinformatics and Systems Biology Program, University of California San Diego 8784, La Jolla, California, USA; 3 Department of Pediatrics, University of California San Diego 8784, La Jolla, California, USA; 4 Novo Nordisk Foundation Center for Biosustainability, Technical University of Denmark 5205, Lyngby, Denmark; Rice University, Houston, Texas, USA

**Keywords:** antibiotic resistance, gene regulation, systems biology, computational biology, RNA-Seq

## Abstract

The earliest responses of pathogenic bacteria to antibiotics can affect the outcome of an infection. While long-term adaptations have been extensively studied, the immediate transcriptional changes that unfold immediately following antibiotic exposure remain poorly understood. Here, we applied iModulon analysis to time-resolved transcriptomic data from *Escherichia coli* exposed to subinhibitory concentrations of two antibiotics (ampicillin and ciprofloxacin), capturing transcriptional regulatory changes occurring within the first 30 min of exposure. This analysis proposes an integrated, three-phase response model: an immediate and sustained primary response that broadly activates stress programs, a transient secondary response that restores redox balance, and a tertiary response that supports long-term survival through metabolic remodeling and antibiotic-specific defenses. These results highlight a coordinated and dynamic regulatory strategy describing how metabolic, redox, and stress responses are integrated to manage the physiological challenges of antibiotic stress. By disentangling these overlapping transcriptional regulatory programs, this work offers a genome-scale understanding of how early regulatory programs are engaged immediately after antibiotic exposure. Together, these findings provide a structured framework for characterizing complex transcriptomic responses and generating testable hypotheses about the regulatory logic that shapes the understudied early phase of antibiotic exposure.

**IMPORTANCE:**

Initial bacterial responses to antibiotics are important for survival and can influence the development of tolerance and resistance. However, this period remains poorly understood, in part, because the transcriptional responses that unfold within minutes of antibiotic exposure are complex and difficult to interpret. In this study, we applied novel data generation and data analytics approaches to resolve the regulatory structure of the initial response of *Escherichia coli* to two antibiotics. We identify a three-phase process that explains how *E. coli* coordinates stress responses, maintains redox homeostasis, and initiates downstream protective programs. The novel transcriptomic analytics elucidate independently regulated sets of genes that constitute cellular processes. By identifying the regulatory modules that change over this initial timescale, we can deconvolute the response based on first principles of cellular physiology.

## INTRODUCTION

The rise of antimicrobial resistance presents an urgent global health crisis, driving the need for deeper insights into how bacteria sense, respond, and adapt to antibiotic exposure (1). Although decades of research have revealed the key aspects of bacterial defense mechanisms, much of our understanding comes from observations made hours after exposure—long after the earliest cellular decisions have shaped the outcome. Despite evidence that *E. coli* can adjust transcription within seconds to minutes of acute stress, very few studies have profiled transcriptome dynamics in the first minutes after antibiotic exposure (2–7). These early moments of antibiotic exposure may hold key insights into how bacteria initiate their defensive responses and transition toward longer-term adaptations, potentially revealing new strategies for therapeutic intervention.

Bacterial responses to antibiotics involve complex, coordinated changes in gene expression across multiple cellular systems (8–12). Traditional transcriptomic analyses have identified diverse stress responses, metabolic adjustments, and resistance mechanisms, but they often struggle to disentangle the transcriptional regulatory networks (TRNs) that orchestrate these changes. Advances in high-quality RNA sequencing and computational methods have enabled systematic approaches to uncover regulatory structure from gene expression data. Among these advancements, iModulon analysis has emerged as a powerful tool for understanding microbial TRNs (13–21). iModulons are independently modulated gene sets, derived from the independent component analysis (ICA) of transcriptomic data. ICA decomposes the gene expression matrix into an iModulon matrix (M) and an activity matrix (A). iModulons, extracted from M, represent groups of genes with coordinated expression patterns that reflect common regulatory control. The activity values in A quantify the strength of each iModulon’s response across conditions. Changes in iModulon activity capture coordinated transcriptional shifts that correspond to specific cellular processes, enabling systematic identification of regulatory programs activated during cellular responses. Unlike traditional differential expression analyses that examine genes individually, iModulons group co-regulated genes into interpretable modules, revealing the coordinated regulatory programs that orchestrate cellular responses with a “top-down” approach. This framework is particularly powerful for understanding how multiple regulatory systems operate simultaneously and dynamically during stress responses, making it well-suited for dissecting the complex cellular response to antibiotics.

In this study, we employ iModulon analysis to investigate the transcriptional landscape of *E. coli* K-12 strain MG1655 during the first 30 min of exposure to sub-inhibitory concentrations of two antibiotics. By capturing gene expression from as early as 1.5 min post-exposure, we characterize a rapid, highly coordinated response that unfolds in three overlapping, yet distinct, phases. The primary response is characterized by an immediate and sustained activation of broad stress-related programs, likely reflecting a generalized effort to stabilize cellular function under sudden antibiotic pressure. As this heightened activity strains the cell’s redox balance, a transient secondary response is triggered to restore homeostasis through temporary activation of anaerobic pathways. Alongside these adjustments, a tertiary response emerges to guide subsequent transcriptional shifts, marked by targeted remodeling of metabolic regulation and the emergence of antibiotic-specific defenses, together helping the cell manage sustained antibiotic stress. These phases operate on overlapping but different timescales and serve distinct purposes and together form a cohesive framework that enables *E. coli* to manage the immediate impact of antibiotics while transitioning toward sustained adaptation. This three-phase process, outlined by iModulons, provides a structured hypothesis for how bacterial regulatory networks orchestrate early responses to antibiotic exposure, capturing the dynamic interplay of stress mitigation and long-term survival strategies. Together, this framework offers a coherent way to interpret early, time-resolved antibiotic responses and motivates testable hypotheses about the regulatory logic that underlies them.

## RESULTS

### iModulons capture gene expression variations caused by antibiotic exposure

**Fig 1:**
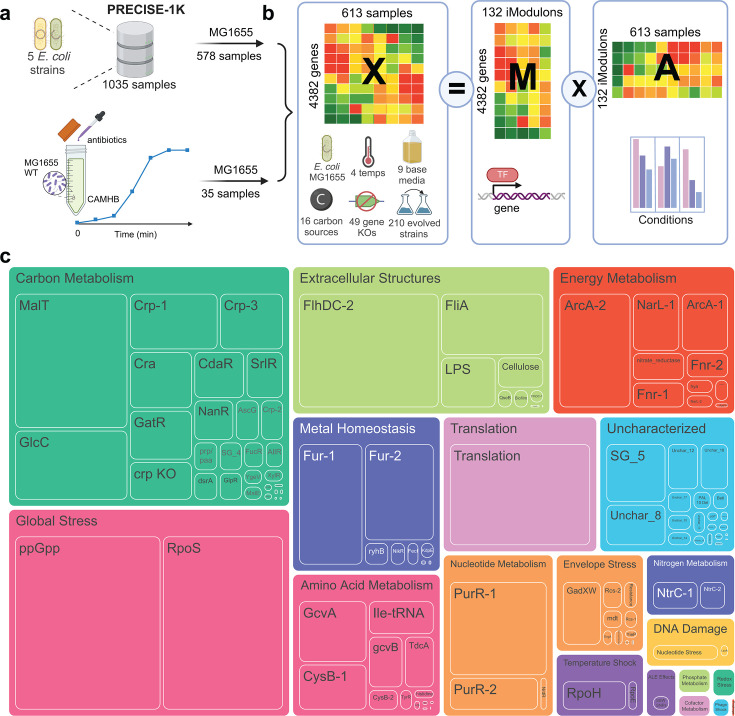
Overview of the data set and iModulon results. (**a**) Data set composition. The data set used in this study combines 578 MG1655 samples from the PRECISE-1K data set and an additional 35 samples from the antibiotics exposure time series generated in this study. (**b**) ICA decomposes the gene expression matrix X into two separate matrices: the iModulon matrix M and the activity matrix A. The columns of the M matrix contain the independent components, from which the gene membership of iModulons is identified. The rows in the A matrix represent the activity of each iModulon across the different conditions in the data set. (**c**) Treemap showing all the iModulons and their assigned functional categories. The size of the boxes represents the variance that the iModulon explains in gene expression in the antibiotic exposure samples. iModulons that are identified as artifacts from data combination and normalization are removed from this figure (see Materials and Methods).

We investigated the transcriptional response of *E. coli* MG1655 to two antibiotics: ampicillin and ciprofloxacin. Ampicillin, a β-lactam antibiotic, inhibits cell wall synthesis, leading to cell lysis, while ciprofloxacin, a fluoroquinolone, targets DNA gyrase and topoisomerase IV, disrupting DNA replication and repair. Gene expression was profiled at 0, 1.5, 3.5, 7.5, 15, and 30 min post-exposure, capturing the earliest transcriptional adjustments. For ampicillin, we examined two concentrations (1/4× MIC and 1/16× MIC) to assess dose-dependent transcriptomic effects. After quality control, 35 antibiotic-treated samples were included in the analysis. To enhance the robustness of our machine learning-based iModulon analysis, these 35 samples were combined with the MG1655 samples from the PRECISE 1K data set (22), and ICA was applied to the combined set of 613 RNA-Seq samples (Fig. 1a and b).

This analysis yielded 132 iModulons, accounting for 84% of global expression variation and 86.9% of the variation in the antibiotic study. Antibiotic exposure triggers diverse regulatory responses represented by iModulons across multiple functional categories. The explained variance of each iModulon can be calculated to quantify its contribution to expression variation in our data set. A treemap of all the iModulons is shown in Fig. 1c, where the box size indicates the explained variance of the iModulon in the antibiotic exposure samples. Stress response iModulons contribute substantially to the expression variation in antibiotic-exposed samples, followed by iModulons associated with various metabolic processes, motility, biofilm formation, and energy metabolism. The full iModulon table with iModulon categories and explained variance can be found in Table S1.

### The response to antibiotics is global and multi-layered

**Fig 2:**
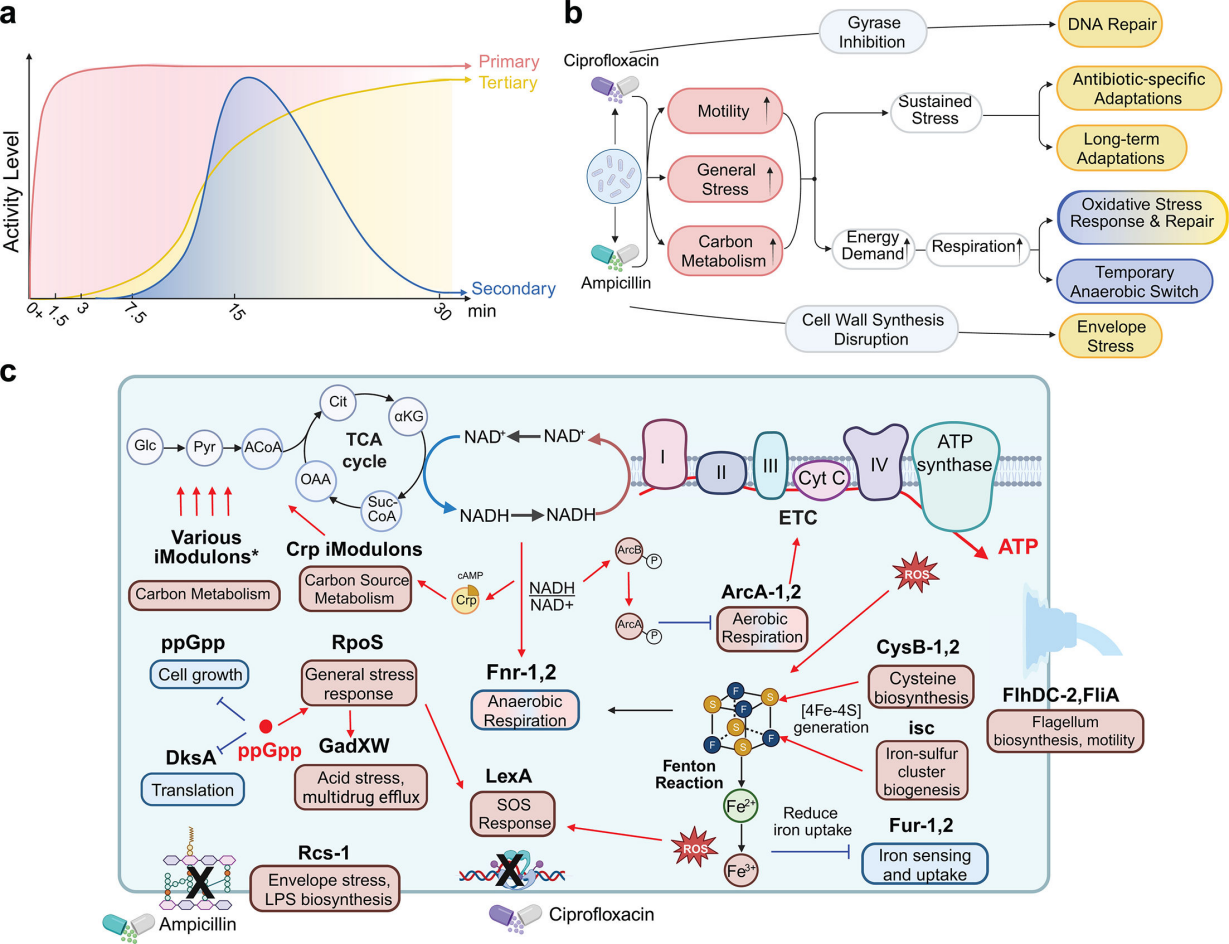
*E. coli’s* early response to antibiotic exposure. (**a**) The diagram outlines the timing and magnitude of the primary, secondary, and tertiary responses of *E. coli* in the first 30 min of antibiotic exposure. The 0+ time point represents samples taken immediately after the antibiotic was added (see Materials and Methods). (**b**) Proposed cascade of cellular events following antibiotic exposure. The colors of the box describing the regulatory responses are consistent with the colors representing primary, secondary, and tertiary responses in panel **a**. (**c**) Major responses in the first 30 min of antibiotic exposure are outlined by key iModulons. The text in the rounded box indicates the cellular function of the associated iModulon. The color of the box displays the relative activity of the iModulon, with red representing overall activation and blue representing overall repression. The phases in which these iModulons are involved can be found in panel b and Fig. S2. The “various iModulons” include the iModulons associated with carbon metabolism that are in Fig. 3*.*

Detailed analysis of iModulon activities reveals that *E. coli*’s early response to antibiotics is rapid, global, and multi-layered. The majority of iModulons exhibit activity changes at different time points following exposure, reflecting a dynamic and phased response. To characterize this process, we categorized iModulons with similar activity patterns and proposed a three-phase structure of the early response (Fig. 2a). These phases are outlined not only by statistical activity correlation between iModulons but also by biological knowledge, detailed in the following sections. The initial transcriptional changes are largely non-specific and occur in two rapid phases: a primary response, which represents an immediate reaction to stress, and a secondary response, which we interpret as a redox consequence of the primary response. The tertiary response, in contrast, reflects a transition toward subsequent transcriptomic remodeling, including antibiotic-specific regulatory changes (Fig. 2b).

**Fig 3:**
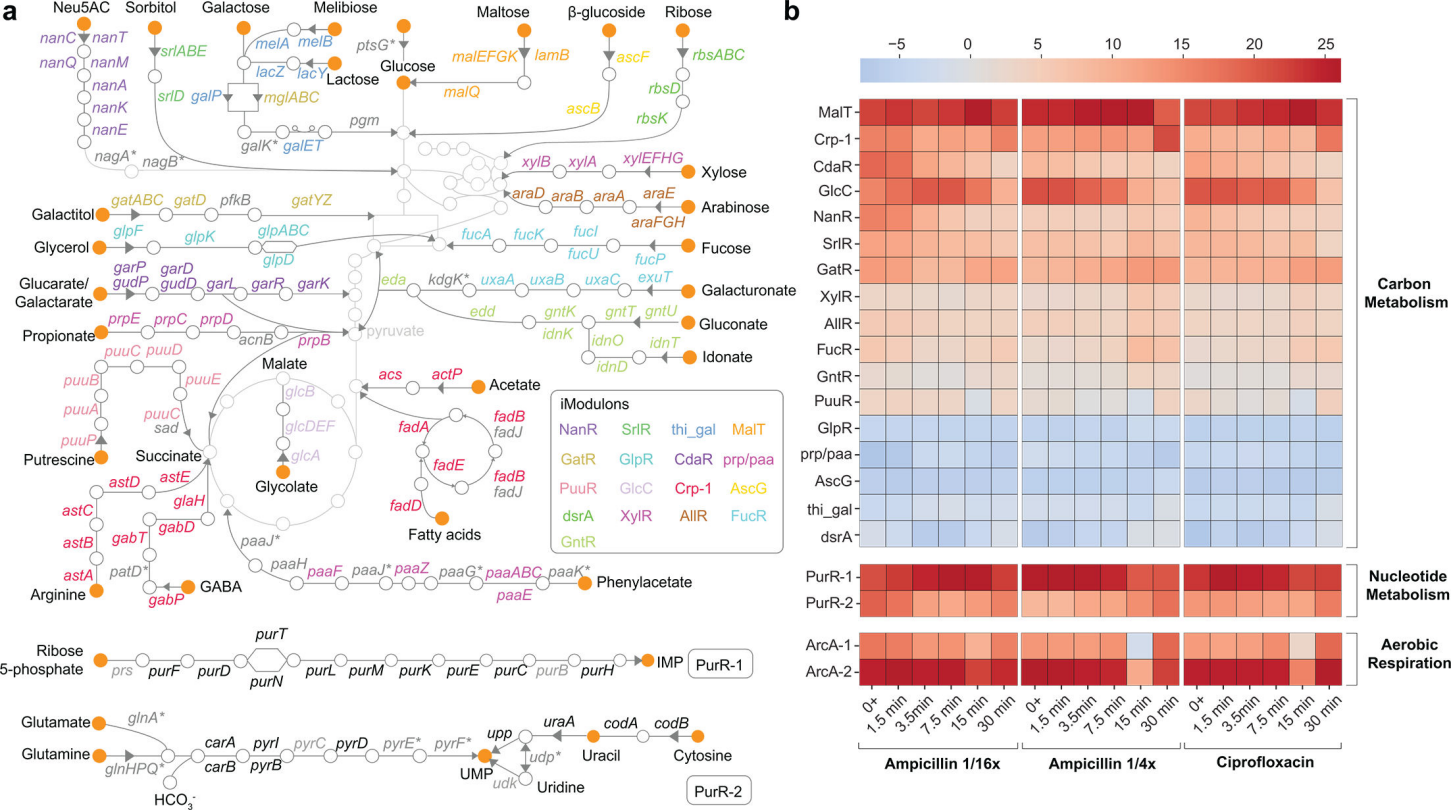
Metabolic remodeling revealed by iModulon activity changes. (**a**) iModulons with significant activity changes upon antibiotic exposure mapped onto *E. coli’s* metabolic network. Gene names are color-coded to distinguish membership in different iModulons. Genes not associated with any iModulon are shown in gray, while genes belonging to iModulons not highlighted in this figure are shown in gray with an asterisk. (**b**) Activity level of carbon and nucleotide metabolism in iModulons, as well as the ArcA iModulons, for the antibiotics-exposed samples.

The primary response occurs almost immediately after antibiotic exposure and involves genes associated with a variety of cellular functions. One of the most pronounced responses is general stress adaptation. The iModulon associated with the global stress response regulator RpoS displays strongly elevated activity. In contrast, we noted that iModulons associated with growth, including the ppGpp iModulon and the Translation iModulon, are downregulated. These observations align with the "fear vs. greed" tradeoff in cellular responses, where cells prioritize stress response over growth under distress (Fig. S1) (23, 24). These three iModulons associated with the fear-greed tradeoff contributed substantially to the gene expression variations in the antibiotic exposure data, accounting for 15.5% of the observed variance in gene expression.

In addition to activating global stress pathways, we observe increased activity in motility-associated iModulons. While resistant *E. coli* strains typically downregulate motility-associated structures to conserve energy (25–27), the early response appears to favor “flight” over “fight.” The high activity of flagella-related iModulons suggests early activation of motility-associated regulatory programs, while iModulons associated with biofilm formation and attachment remain repressed. This prioritization of motility over sessile adaptations may reflect an initial survival strategy, enabling the cells to evade antibiotic stress before committing to long-term resistance mechanisms.

iModulon analysis also reveals rapid changes in the expression of genes associated with cellular structures, metal homeostasis, and, notably, metabolism. The activity of all the iModulons for the antibiotic-treated samples can be found in Fig. S2, and discussions on other interesting iModulons from the primary response can also be found in Note S1. Given that the early responses outlined by the iModulons mentioned above would impose substantial energetic demands on the cell, we observe widespread metabolic shifts accompanying these responses, emerging as another fundamental aspect of the primary response. These metabolic changes will be explored next before we turn to the secondary and tertiary phases, where the transcriptional landscape evolves and becomes more specialized and adaptive. The major transcriptional changes outlined by key iModulons are presented in Fig. 2c and will be discussed in detail in the following sections.

### iModulons uncover detailed metabolic remodeling

We propose that increased energy demands and a need to maintain metabolic flexibility under stress prompt *E. coli* to undergo extensive metabolic shifts as a primary response to antibiotic exposure. In our data set, iModulons associated with carbon metabolism and nucleotide biosynthesis show prominent reprogramming, accompanied by increased activity in iModulons linked to aerobic respiration and energy production.

The cells appear to be programming their metabolism to access available nutrients, as evidenced by increased activity in iModulons related to alternative carbon source utilization and anaplerosis. These iModulons are enriched in carbon source transporters and pathways that feed different carbon sources into glycolysis and the TCA cycle. Fig. 3a maps these iModulons onto carbon metabolic pathways and illustrates how they facilitate the incorporation of different carbon sources into central carbon metabolism. In contrast, iModulons associated with certain carbon sources are downregulated (Fig. 3b). This includes β-glucosides, which wild-type *E. coli* cannot metabolize, as well as other carbon sources that may be deprioritized due to differences in utilization preferences (28). Since the medium used in this study is not known to be carbon-rich, and we do not observe hierarchy in the activation of these metabolic iModulons, we interpret the activation of alternative carbon utilization as an increase in *E. coli*’s metabolic flexibility, supporting a steady supply of energy and metabolic intermediates by accessing multiple carbon sources. Several iModulons associated with the cyclic AMP receptor protein (CRP) further contribute to this adaptability by linking central carbon metabolism to processes such as fatty acid and aromatic compound degradation, as well as nitrogen and phosphate metabolism. This metabolic flexibility, as evidenced by broad activity shifts across metabolic iModulons, might help *E. coli* sustain metabolic flux despite stress-induced disruptions while efficiently adjusting to changing nutrient availability*.*

Simultaneously, iModulons associated with nucleotide metabolism and linked processes are upregulated, reflecting an increased demand for nucleotides (Fig. 3a and b). This upregulation could be supporting essential processes such as DNA repair, replication, and stress responses, as well as providing key metabolic regulators and signaling molecules. Additionally, antibiotic treatment can disrupt nucleotide pools and deplete building blocks, prompting the upregulation of nucleotide synthesis pathways in order to compensate (29–31).

The active central metabolism fuels a surge in cellular respiration and increased energy production, supported by the high activity of the ArcA iModulons (Fig. 3b). They are enriched in genes associated with the TCA cycle, oxidative phosphorylation, the electron transport chain, and other key processes in aerobic respiration (Tables S2 and S3). While these early metabolic responses allow the cell to cope with immediate stress, they also lead to the accumulation of toxic byproducts, including reactive oxygen species (ROS) (32, 33). ROS can compromise iron-sulfur clusters via the Fenton reaction, thereby producing highly reactive hydroxyl radicals that further damage cellular components. The Fur iModulons related to iron uptake show significantly reduced activities, likely as a response to increased intracellular free iron generated by the Fenton reaction (Fig. S2). Therefore, we postulate that while the primary response facilitates rapid adaptation to sudden stress, it also imposes substantial burdens and is unsustainable over time. Ultimately, the cell must adopt more sustainable strategies to manage the effects of sustained stress and support survival (34, 35).

### iModulon dynamics reveal transient redox regulation and anaerobic shift

**Fig 4:**
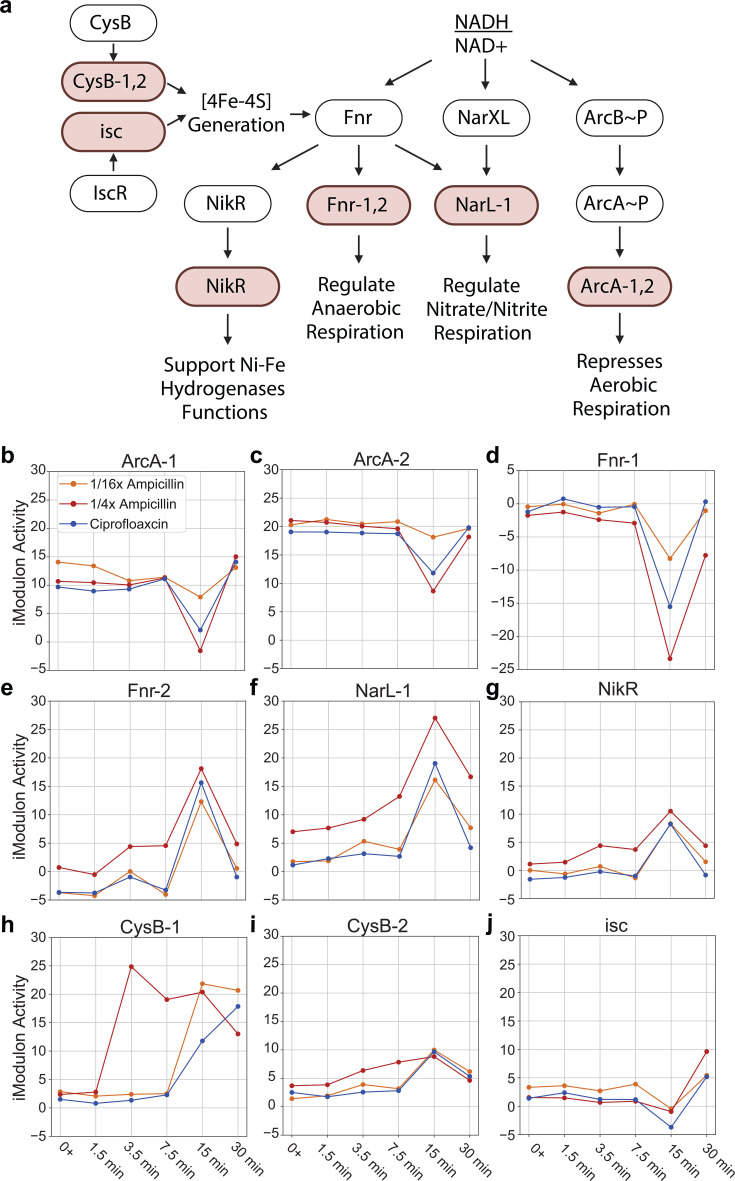
Key iModulons display temporal activity patterns. (**a**) Mechanistic outline of the secondary response with the associated regulators and iModulons. The colored boxes represent iModulons, and the black and white boxes represent regulators. (**b–j**) iModulons associated with secondary response show significant activity changes over time. Note that genes in the Fnr-2 iModulons are negatively weighted. Therefore, an increase in iModulon activity reflects a “more significant decrease” in gene expression (20)*.*

The observed global activation of carbon utilization iModulons in the primary response enables the cell to rapidly generate energy through aerobic respiration to cope with antibiotic stress. However, this increased metabolic activity can disrupt redox balance, as redox cofactors such as NADH accumulate when glucose consumption outpaces the cell’s capacity to reoxidize reduced equivalents (36). Specifically, the increased flux through the TCA cycle produces NADH faster than the electron transport chain can regenerate NAD+, leading to an elevated NADH/NAD+ ratio. This redox imbalance triggers redox-sensing regulators to adjust gene expression and restore redox homeostasis (37, 38). In this context, several iModulons governed by key regulators that are sensitive to redox signals—including ArcA, Fnr, and Crp—exhibit sharp activity changes by 15 min post-exposure. We hypothesize that this transient shift, which stabilizes later for some iModulons, could represent a “redox reset” to counteract the redox disturbances induced by the primary response (Fig. 4a).

A high NADH/NAD+ ratio triggers the phosphorylation of the ArcAB system, leading to ArcA-mediated repression of aerobic respiration genes (39). Accordingly, we observed a sharp decrease in ArcA iModulon activities at 15 min (Fig. 4b and c). At the same time, iModulons associated with anaerobic metabolism increased in activity, including genes regulated by Fnr and those involved in alternative electron acceptor utilization, such as dimethyl sulfoxide (DMSO) and nitrite respiration controlled by NarLX (Fig. 4d through f). The genes in these iModulons are shown in Tables S4 to S6. This shift might provide an alternative route for NADH reoxidation to NAD+, thereby restoring redox balance. The activation of Fnr also upregulates the NikR iModulon (Fig. 4g), which includes the NikABCDE nickel transporter essential for NiFe hydrogenase synthesis during anaerobic growth (40, 41). We also detected increased activity in the cysteine iModulons (Fig. 4h and i) and later activation of the *isc* iModulon at 30 min (Fig. 4j), likely supporting iron-sulfur cluster biogenesis to maintain Fnr function during this phase (42, 43). While this response resembles overflow metabolism (38), we did not observe significant changes in genes associated with acetate or lactate production. It is possible that these pathways were transiently activated between 15 and 30 min but were not captured in our samples.

Importantly, the secondary response does not appear to be sustained, suggesting that its primary function may be to correct redox imbalance rather than to serve as a long-term metabolic adaptation. Once the electron transport chain catches up or additional regulatory mechanisms adjust metabolism, the cells likely shift back to aerobic pathways to maximize energy efficiency. This observed transient activation of anaerobic pathways in our data set has not been emphasized in prior studies of *E. coli* antibiotic responses to our knowledge. This distinct and dynamic shift warrants further investigations to clarify the metabolic and regulatory mechanisms that may underlie it and its role in antibiotic adaptation.

### Tertiary response: shift to long-term adaptation

As the stress continues, the cell appears to transition from short-term corrections to adjustments supporting survival under sustained antibiotic exposure. While the primary response is observed to remain active throughout the time course and the secondary response addresses redox imbalances, we observe gradual shifts in the activity of many iModulons beginning around 7.5 min and persisting through 30 min, which are consistent with broad regulatory adjustments in the tertiary response. Many of the iModulons activated in the primary response evolve in this time frame. These ongoing adjustments contribute to the broader regulatory shifts that define the tertiary response and highlight how *E. coli* may fine-tune its initial defenses to meet the demands of prolonged antibiotic stress. Although these changes are distributed across a wide range of pathways (Fig. S2), one notable example is the progressive increase in Crp-related iModulons, particularly Crp-2, which is enriched in transcriptional regulators of various carbon utilization pathways (Table S7).

Prolonged stress appears to shift metabolic regulation from broad activation toward a more selective strategy. While many alternative carbon utilization iModulons remain active, certain iModulons linked to specific carbon sources—such as CdaR (glucarate/galactarate metabolism), GlcC (glycolate conversion to malate), and NanR (feeding Neu5Ac into glycolysis)—gradually decrease in activity (Fig. S2). This selective downregulation may reflect a refinement of metabolic priorities as adaptation progresses, potentially influenced by constraints such as resource allocation, metabolic efficiency, or regulatory burden. Meanwhile, Crp-2 and its associated regulators, several of which govern carbon-associated iModulons activated during the primary response, may guide this transition from global carbon activation to more coordinated, Crp-mediated metabolic remodeling. Given Crp’s role as a master regulator that integrates metabolic and redox signals, its increasing activity may contribute to balancing carbon utilization with the cell’s overall redox state under sustained stress.

### iModulons identify antibiotic-specific response

The tertiary response also includes antibiotic-specific adaptations that could reflect the cell’s efforts to counteract different types of antibiotic stress. The antibiotics used in this study have distinct mechanisms of action: ampicillin, a β-lactam, disrupts peptidoglycan synthesis, while ciprofloxacin, a fluoroquinolone, interferes with DNA replication by inhibiting DNA gyrase and topoisomerase IV. Consistent with these differences, we identified specific iModulons that capture unique aspects of each antibiotic’s impact on *E. coli*.

**Fig 5:**
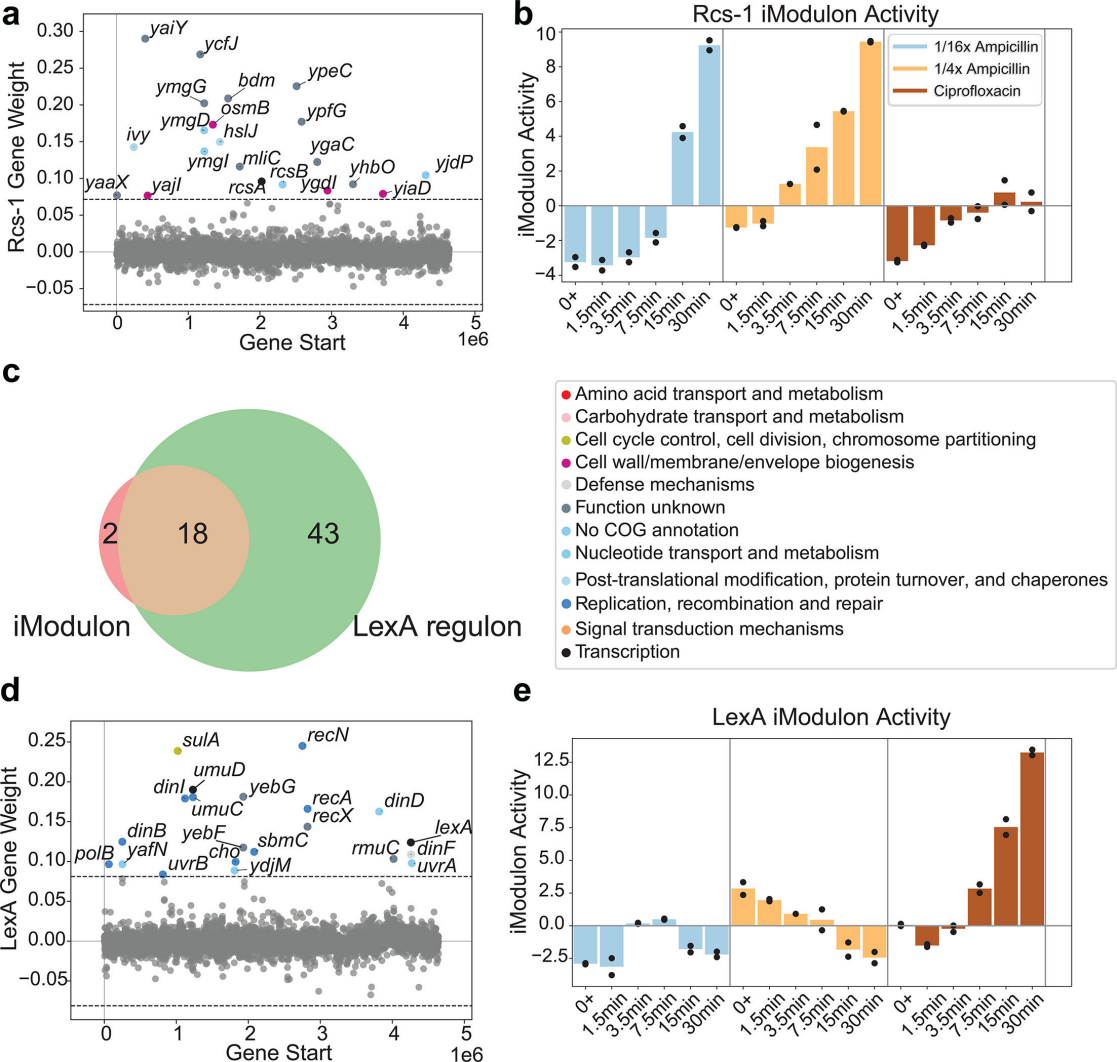
Antibiotic-specific iModulon responses. (**a**) iModulon gene membership for the Rcs-1 iModulon. (**b**) The activity of the Rcs-1 iModulon for antibiotic-treated samples. (**c**) Venn diagram showing the overlap in gene membership between the LexA iModulon and the Lex A regulon. (**d**) iModulon gene membership for the LexA iModulon. (**e**) The activity of the LexA iModulon for antibiotic-treated samples.

*E. coli* relies on multiple systems to manage envelope stress, each tailored to specific types of damage (44). While many of these systems are represented in our iModulon analysis (Fig. S2), the Rcs system exhibited the most prominent signal in response to ampicillin exposure. The Rcs system (regulator of capsule synthesis) responds to disruptions in peptidoglycan biosynthesis and plays a crucial role in resistance to β-lactams (45). We identified two Rcs-related iModulons, with Rcs-1 exhibiting rapid and sustained activation following ampicillin exposure. Although Rcs-1 only partially overlaps with the known Rcs regulon, it includes *rcsA*, *rcsB*, and several genes linked to envelope stress, such as *ivy* and *mliC* (lysozyme inhibitors) (46), *osmB* (osmotic stress resistance), *hsiJ* (novobiocin resistance), and *ygaC* (pH stress response) (Fig. 5a) (47). Notably, 14 of its 21 genes are uncharacterized y-genes, many of which are previously proposed to contribute to envelope biogenesis and have been associated with antibiotic responses (45). We performed MIC measurements using knockout strains from the Keio collection for 17 genes in this iModulon (the remaining four are not represented in the collection). Most knockouts exhibited MICs comparable to the wild type or showed slightly increased susceptibility to ampicillin, while two showed modest increases in resistance (Fig. S3). Interestingly, deletion of the highest-weighted gene in this iModulon, *yaiY*, led to a notable reduction in MIC, suggesting increased vulnerability. This, combined with strong early activation of Rcs-1, supports its potential role in immediate defense against β-lactam-induced envelope stress (Fig. 5b), making it a promising target for further investigations.

The Rcs-2 iModulon, in contrast, is enriched with well-characterized Rcs-regulated genes, including the *wca* and *cps* genes for colanic acid biosynthesis and the *yjbEFGH* genes for exopolysaccharide production (Fig. 5c) (48). While its activation is also ampicillin-specific, the response is slower and less pronounced compared to Rcs-1 (Fig. 5d). This temporal difference could be interpreted as colanic acid biosynthesis serving as a secondary adaptation to reinforce the envelope over time. Meanwhile, the rapid activation of Rcs-1 may reflect transcriptional responses associated with immediate envelope destabilization, potentially indicating a prioritization of acute responses followed by longer-term structural adaptations. These nuanced behaviors we observe in our data set could reflect the functional complexity of the Rcs system and present opportunities for further investigations.

Ciprofloxacin inhibits DNA gyrase, causing DNA stress that activates the SOS response. Accumulated single-stranded DNA (ssDNA) triggers RecA, which promotes LexA self-cleavage and derepresses SOS genes (49). In response to ciprofloxacin, we identified a LexA-associated iModulon comprising many DNA damage-inducible genes from the LexA regulon (Fig. 5e and f), including *lexA*, *uvrA*, *uvrB* (nucleotide excision repair), *recA*, *recN* (homologous recombination), *sulA* (cell division inhibition), and the error-prone polymerases *polB*, *dinB*, and *umuCD* (translesion synthesis). The activity of this iModulon increases after 7.5 min of ciprofloxacin exposure, likely reflecting the accumulation of ssDNA beyond the activation threshold for the SOS response and remains elevated, consistent with ongoing DNA damage from DNA gyrase inhibition. In contrast, this iModulon stays inactive during ampicillin treatment (Fig. 5g), likely because β-lactams cause DNA damage indirectly through the DpiBA system, leading to a slower and less severe SOS activation (50). Ciprofloxacin’s direct disruption of DNA appears to rapidly induce the full SOS program captured by this iModulon, reflecting the magnitude and urgency of its DNA-targeting mechanism. Another ciprofloxacin-specific but less pronounced signal is discussed in Note S2.

### Differential iModulon activities reveal dose-dependent transcriptomic responses

To assess whether transcriptional responses vary with antibiotic dosage, we compared *E. coli*’s activity under two subinhibitory concentrations of ampicillin: 1/4× MIC and 1/16× MIC. As reflected in the preceding sections, the overall regulatory and metabolic responses were largely similar, indicating that the core regulatory and metabolic responses to β-lactam stress are independent of dose at subinhibitory concentrations (Fig. 1d and 3b). However, we find that higher concentrations tend to drive stronger shifts in iModulon activity, which supports the interpretation that dosage primarily affects the magnitude of adaptation rather than its regulatory structure (Fig. 4a through f; Note S3; Fig. S4). Notably, we observed selective activation of the TdcA iModulon only at the higher concentration. This iModulon contains genes in the *tdc* operon, which support threonine and serine transport and metabolism during anaerobiosis. Similarly, the hya iModulon enriched in hydrogenase 1 genes is also activated around 15 min only for 1/4× MIC (Note S3; Fig. S4). Differential iModulon activity analysis is carried out using the approach outlined in Materials and Methods. The activation of these iModulons at higher antibiotic concentrations could indicate that greater stress intensifies the cell’s metabolic demands or the anaerobic-like redox reset, possibly leading to the engagement of additional metabolic pathways.

## DISCUSSION

Bacterial survival under antibiotic stress relies on complex, multilayered adaptations. In this study, we applied iModulon analysis to high-resolution time-series RNA-seq data to capture the early transcriptional response of *E. coli* to subinhibitory antibiotic exposure. Our work provides a computational-based framework for examining the changes in the transcriptome during this underexplored early period of antibiotic exposure and offers a hypothesis for the regulatory sequence that may unfold within the first 30 min. From the iModulon activity patterns, we outline a structured, three-phase process of response. This proposed structure provides a genome-scale view of how regulatory programs may be dynamically organized over time, offering new insight into the early transcriptional responses of *E. coli* under antibiotic pressure.

Our analysis suggests a three-phase dynamic response that may reveal how *E. coli* manages the competing demands of antibiotic stress through a coordinated, layered strategy, with each layer addressing distinct challenges. The primary response provides a rapid, broad activation of stress responses to stabilize cellular function under sudden pressure. This global response is energetically demanding and disrupts redox balance, triggering a transient secondary response that restores homeostasis through anaerobic pathways. Meanwhile, the tertiary response establishes more sustainable adaptations, such as metabolic remodeling and antibiotic-specific defenses, to support prolonged survival as stress persists. Importantly, the responses are not strictly sequential; the sustained activity of the primary response alongside the emergence of tertiary adaptations illustrates how *E. coli* layers its defenses, maintaining immediate survival mechanisms while gradually developing more specialized, long-term strategies. Some iModulons contribute to multiple phases, with their activities dynamically shifting to support this transition and reflect the developing demands of ongoing antibiotic stress.

Beyond proposing this general framework, our analysis uncovers regulatory features that merit further investigation. The strong, ampicillin-specific activation of the Rcs-1 iModulon points to an envelope stress response enriched with largely uncharacterized *y*-genes, suggesting potential roles in β-lactam tolerance that remain unexplored. The identification of previously unannotated genes in a key antibiotic response pathway provides an opportunity to refine functional annotations and potentially identify biomarkers for emerging resistance. Additionally, the selective activation of the *tdc* operon and *hya* genes at higher ampicillin concentrations—one of the few dose-dependent differences observed—may indicate an additional layer of metabolic adaptation under more severe or prolonged stress, potentially linked to sustained anaerobic-like conditions.

Together, these findings offer a new perspective on the early stages of bacterial adaptation to antibiotics. By dissecting transcriptional complexity into a structured, three-phase process, this work offers a data-driven framework for interpreting how *E. coli* coordinates multiple regulatory programs as stress unfolds. Beyond outlining this progression, our results also connect the underlying biological processes that drive each phase, shedding light on potential interactions among metabolic, redox, and stress-response pathways during acute antibiotic exposure. These connections also highlight specific regulatory and metabolic features that give rise to grounded, targeted, and testable hypotheses, including Rcs-linked envelope remodeling, transient redox adjustments, and the dose-dependent activation of anaerobic and metabolic pathways that could be examined experimentally in future work. Future studies can assess how this phased framework applies across different classes of antibiotics, strains (including clinical isolates), and longer timeframes, providing a bridge between the immediate transcriptional responses captured here and the well-documented adaptations that emerge at later stages. Integrating transcriptomic data with other data types such as proteomics, metabolomics, or pangenomics may further illuminate the biological determinants that shape early regulatory dynamics in *E. coli*. Although developed in the context of antibiotic stress, this framework may also help clarify temporal structure and progression in other rapidly evolving or multi-layered cellular responses, providing a generalizable strategy for interpreting complex time-resolved transcriptomic data.

## MATERIALS AND METHODS

### Culture conditions and sample preparation

An overnight *E. coli* MG1655 culture was inoculated from a glycerol stock and grown at 37°C with shaking. The next morning, a fresh culture in MHB-CA was prepared at an initial OD₆₀₀ of 0.05. Once the OD₆₀₀ reached 0.5, the cultures were aliquoted into plastic tubes (16 mL per tube) containing stir bars and placed on a 24-well magnetic heat block. Ampicillin (final concentration: 16 µg/mL for 1/4× MIC or 4 µg/mL for 1/16× MIC) or ciprofloxacin (0.016 µg/mL for 1/4× MIC) was immediately added. Concentrations of 1/4× MIC and 1/16× MIC were chosen because they represent sub-inhibitory levels that elicit transcriptional responses without causing immediate growth inhibition or cell lysis. Samples were collected in duplicate at 0, 1.5, 3.5, 7.5, 15, and 30 min post-exposure, treated with RNAprotect Bacteria Reagent, and stored at −80°C for RNA-seq library preparation. Because cultures were transferred into fresh medium during mid-exponential growth and sampled over only a 30-min period, no growth-phase transitions were expected during the experiment. This assumption is supported by the fact that the iModulon activity profiles observed in this study differ from those associated with stationary-phase entry described in prior work (51).

### RNA extraction and library preparation

Total RNA was extracted using a Luna Nanotech PuroMAG Total RNA Purification Kit (NKM051-384), which included a DNase I treatment and a modification to incorporate bead beating as a means of cell lysis. RNA quality and concentration were assessed using an Agilent TapeStation and a Nanodrop, respectively. One microgram of total RNA underwent ribosomal RNA removal using RiboRid (52), and the resulting rRNA-depleted RNA was used to make RNA-seq libraries with the Kapa Biosystems (Roche) RNA HyperPrep Kit, following the manufacturer’s protocol.

### Data preprocessing

Quality control of the antibiotic exposure data set was performed following step_3 of the iModulonMiner workflow (53). One sample was filtered out during quality control (ampicillin 1/4× MIC 7.5 min) due to the low number of mapped reads and poor replicate correlation. To improve the quality of the ICA decomposition with more data, we combined our antibiotic-treated MG1655 samples with MG1655 expression data from the PRECISE 1K compendium (22). Specifically, we filtered the PRECISE 1K data set to retain only samples from strain MG1655, resulting in 578 samples that were merged with our data set prior to normalization. All samples were normalized to a project reference.

### Data normalization and control sample evaluation

In the antibiotic exposure data set, the 0 min time points were collected immediately after antibiotic addition (0+). Including handling and processing time, the samples may already capture the earliest transcriptional changes. To provide an antibiotic-free baseline for normalization, we used CAMHB no-antibiotic control samples from the PRECISE 1K data set (54), which were generated under the same experimental conditions, by the same researcher, and using the same experimental protocol. The PRECISE 1K study has previously demonstrated that control samples produced under these conditions show highly consistent gene expression profiles and iModulon activities (22), supporting their use as a reliable reference.

Recent analyses on the updated iModulonDB platform show that for the samples from Sastry’s study, most iModulons exhibit consistent activity across samples from different projects conducted under the same protocol (21, 22). The CSP iModulon shows higher activity levels out of consistency, and for this reason, it was excluded from downstream analysis in this study.

We additionally generated an alternative iModulon structure using the average of the 0+ samples from the antibiotic exposure data set as the normalization reference. This alternative approach produced highly consistent iModulon structures (overall Pearson *R* correlation > 90%) and activity trends, with strong agreement particularly in the secondary and tertiary phases of the response. However, the 0+ samples may already capture immediate gene expression shifts. When using them as the reference, these early signals are effectively subtracted out during normalization, reducing the ability to detect the full extent of primary response dynamics. For this reason, we selected the PRECISE 1K CAMHB controls as the reference for our primary analysis. The alternative iModulon structure is available in our GitHub repository for reference.

### Extracting and characterizing iModulons

ICA calculations and iModulon extractions were performed following the iModulonMiner workflow (53). The optimal dimensionality of the data set was determined to be 200, resulting in 132 iModulons (55). iModulon enrichments against the known regulons from RegulonDB and metabolic pathways from Kyoto Encyclopedia of Genes and Genomes (KEGG) as well as explained variance calculations were conducted using the PyModulon Python package. iModulons are named with the associated regulator or inspected individually and named based on their gene content and functional enrichment results.

The iModulons selected for detailed interpretation were chosen using a combination of quantitative and biological criteria. We considered the amount of variance explained by each iModulon and assessed the consistency of its activity patterns across biological replicates. In addition, we prioritized modules with clear biological relevance or known involvement in stress responses, while uncharacterized modules were noted but interpreted more cautiously. Similarity in biological function and temporal behavior was also taken into account when grouping or comparing iModulons. This approach ensured that both the statistical structure of the data and the biological interpretability of each module informed the analyses presented in the main text.

To define the three-phase response framework, we integrated statistical measures that grouped iModulons with similar activity profiles together with biological knowledge about the underlying regulons and pathways. Incorporating both types of information was necessary, as clustering based solely on activity patterns often produced groupings too coarse to capture the full complexity of the transcriptional response.

### Clarification on iModulon artifacts

Since our data set combines the PRECISE 1K and antibiotic exposure time series data, we identified a small number of iModulons with high variance that appear to result from technical differences between data sets rather than meaningful biological activity. The X matrix in PRECISE 1K contains 4257 genes, while the antibiotic exposure series has 4,305 genes. iModulons Unchar_2 consists exclusively of the gene differences between these two data sets. It does not reflect a biological signal and was therefore excluded from the downstream analysis to ensure that our interpretations focus on genuine regulatory signals. In addition, we removed the csp iModulons whose activity patterns are likely influenced by specific samples in the data set and do not reflect consistent or biologically interpretable responses. By filtering out these artifacts, we aimed to maintain a robust and reliable analysis of the transcriptional adaptations to antibiotic stress.

### Differential iModulon activity analysis

To evaluate the differences in iModulon activity between two conditions, we first characterized the variability of each iModulon across biological replicates. For every iModulon, the distribution of replicate-to-replicate activity differences was computed and fit with a log-normal probability model. This replicate-derived distribution served as the null expectation for that iModulon.

For each pairwise comparison, the mean activity within each condition was calculated (when replicates were available), and the absolute difference between these means was obtained. This observed difference was then assessed against the corresponding log-normal distribution to determine a *P*-value for that iModulon. Multiple comparisons were corrected using the Benjamini–Hochberg procedure. iModulons were considered to show significant differential activity when the absolute activity difference exceeded five units and the FDR-adjusted *P*-value was below 0.01. Differential activity between two conditions can be visualized through differential iModulon activity plots, which display the activity shifts across all iModulons.

## Data Availability

The RNA-Seq data generated in this study have been deposited to the Gene Expression Omnibus (GEO) with the accession number GSE295494. All code and data used to generate the results in this paper can be found on GitHub (https://github.com/AnnieYuan21/antibiotICA-MG1655). The general iModulon analysis pipeline can be found at https://github.com/SBRG/iModulonMiner.

## SUPPLEMENTAL MATERIAL

Supplemental Information msystems.01584-25-s0001.pdf Notes S1 to S3, Fig. S1 to S6, and supplemental table captions.

Table S1 msystems.01584-25-s0002.csv iModulon table.

Table S2 msystems.01584-25-s0003.csv ArcA-1 iModulon gene composition.

Table S3 msystems.01584-25-s0004.csv ArcA-2 iModulon gene composition.

Table S4 msystems.01584-25-s0005.csv Fnr-1 iModulon gene composition.

Table S5 msystems.01584-25-s0006.csv Fnr-2 iModulon gene composition.

Table S6 msystems.01584-25-s0007.csv NarL-1 iModulon gene composition.

Table S7 msystems.01584-25-s0008.csv Crp-2 iModulon gene composition.

ASM does not own the copyrights to Supplemental Material that may be linked to, or accessed through, an article. The authors have granted ASM a non-exclusive, world-wide license to publish the Supplemental Material files. Please contact the corresponding author directly for reuse.
